# 2-[(4-Hydroxybenzyl) Amino] Phenol (HBAP) Restores the Mutated p53 to the Level Similar to That of Wild-Type p53 Protein and Inhibits Breast Cancer Growth *in vivo* to by Inducing Tumor Cells Apoptosis

**DOI:** 10.3389/fcell.2020.574799

**Published:** 2020-11-26

**Authors:** Chenxi Xu, Jianjian Zhuang, Xiaobo Zhang

**Affiliations:** College of Life Sciences, Zhejiang University, Hangzhou, China

**Keywords:** cancer cells, apoptosis, compound, deep sea, mutated p53

## Abstract

P53 is a transcriptional factor that plays important roles in apoptosis and is mutated in more than 50% of tumor cells. However, the restoration of mutated p53 to the level similar to wild-type p53 by a natural compound has not been explored intensively. In this study, the 2-[(4-hydroxybenzyl) amino] phenol (HBAP) compound, obtained from deep-sea virus-challenged thermophile *Geobacillus* sp. E263, interacted specifically with the mutated p53 protein. HBAP was able to induce apoptosis of p53-mutated breast cancer cells, but not normal breast cells and p53-unmutated breast cancer cells. HBAP activated the mutant p53 transcriptional activity by restoring the function of mutant p53 to that of wild-type p53. Further analysis indicated that HBAP bound only to the DNA binding domain of mutant p53 and that the interaction was dependent on the HBAP hydroxyl groups. *In vivo* data demonstrated that HBAP was toxicity-free and could suppress tumor growth by inducing tumor cell apoptosis. Therefore our findings revealed that recovering mutated p53 function to that of wild-type p53 caused by HBAP triggered cancer cell apoptosis and that metabolites from deep-sea virus-challenged thermophiles could be a promising source of anti-tumor drugs.

## Introduction

Tumor suppressor p53 protein has been known to play very important roles in preventing tumor progression (Kastan et al., 1991; Yang et al., 2017). P53 is a transcriptional factor that can regulate the expression of a wide variety of genes involved in cell cycle arrest and apoptosis (Bieging et al., 2014). As previously reported, p21 is a CDK (cyclin-dependent kinase) inhibitory protein transcriptionally regulated by p53, whose activation leads to the cell cycle arrest in the G1 phase (Li et al., 1994; Martin et al., 2016). PUMA (p53 upregulated modulator of apoptosis) is a protein transcriptionally activated by p53 that can bind to Bcl2 located in the mitochondria to induce cytochrome c release, thus resulting in apoptosis (Vousden, 2005; Zhang et al., 2016). The p53 protein consists of three domains, the N-terminal transactivation domain (NTD), the central DNA-binding domain (DBD), and the carboxyl-terminal oligomerization and regulatory domain (CTD) (Bieging et al., 2014). In cells, the p53 protein forms a tetramer through the CTD domain (Bieging et al., 2014) and the DBD domain of p53 is critical for p53 function, as it binds to specific response elements (Bao et al., 2017). Mutations in p53 are observed in more than 50% of cancers (Hollstein et al., 1994; Hollstein et al., 1997; Miller et al., 2019) and most of them are localized in DBD (Pradhan et al., 2019). In this domain, there are six “hot spot” residues that undergo mutations with exceptionally high frequency that can be categorized into contact and structural mutations (Liu et al., 2009). Contact mutations alter residues that are important for DNA binding, while structural mutations change the three-dimensional folding of DBD domain (Liu et al., 2009). The mutant p53 not only exerts dominant-negative effect on wild-type protein, but also displays gain-of-function properties (Adams et al., 2011), such as contributing to the malignant tumor progression (Hollstein et al., 1997; Muller and Vousden, 2014). Rehabilitation of p53 function *in vivo* has been reported to lead to tumor regression (Venkatanarayan et al., 2016). Therefore the restoration of mutant p53 function to that of wild-type p53 has attracted increasing attention in recent years.

Many strategies have been introduced to retrieve the structure and function of mutant p53 to similar to those of wild-type p53, including small peptides, such as CDB3, and small compounds, such as CP-31398, RITA, Nutlin and NSC31926 (Oren et al., 2016). CDB3 is a nine-residue peptide that can bind to p53 DBD domain to stabilize the p53 protein *in vitro* (Friedler et al., 2002). CP-31398 and RITA can bind to p53 to stabilize the structure of p53 mutants (Issaeva et al., 2004; Tang et al., 2007), while Nutlin prevents the MDM2 (murine double minute 2) from binding to p53 to increase the p53 level in cells (Joseph et al., 2010). NSC31926 is a metal ion chelator that can restore the R175H p53 mutant structure to wild-type p53. This effect is possibly achieved by NSC31926 increasing the zinc bioavailability for the mutant p53, due to wild-type p53 needs Zn^2+^ to fold correctly (Yu et al., 2012). Recently, the compound NSC59984 has been reported to restore p53 signaling pathway by activating p73 activity (Zhang et al., 2015). Currently, compounds that can restore mutant p53 to the wild-type are mostly artificially synthesized. Knowledge about natural products from deep-sea microorganisms that have this effect is still limited.

In deep-sea hydrothermal vent ecosystems, viruses regulate microbial diversity and abundance (He et al., 2017). During the process of tumor development, the normal metabolic process of tumor cells is disturbed due to the cancer cell massive proliferation and the need for a large amount of nutrients (Flores et al., 2013). This metabolism disturbance occurs in a similar way in host bacteria infected by phages, so that the infected bacteria begin to produce small molecular compounds resistant to phage infection (Ankrah et al., 2014; Zhang, 2019). Thus, we hypothesized that metabolites produced during the interaction between phages and host bacteria could be an important source of antitumor compounds. To obtain the natural products from deep-sea organism capable of restoring mutant p53 to the wild-type, the thermophile *Geobacillus* sp. E263, isolated from a deep-sea hydrothermal vent (Jin et al., 2015), was challenged by its bacteriophage GVE2 in this study. The results showed that the compound 2-[(4-hydroxybenzyl) amino] phenol (HBAP), derived from GVE2-infected *Geobacillus* sp. E263, can retrieve the mutant p53 function (p53^R280K^) to that of wild-typep53, leading to breast cancer cell apoptosis.

## Materials and Methods

### Cell Culture

Human non-small cell lung cancer cells (H1299) and human breast cancer cells (MCF-7) were grown in DMEM medium (Dulbecco’s modified eagle medium) (Gibco, United States) supplemented with 10% FBS (fetal bovine serum) (Gibco, United States). Human breast cancer cells MDA-MB-231 and MDA-MB-468 were cultured in Leibovitz’s L-15 medium (Gibco, United States) supplemented with 10% FBS. Human breast non-carcinoma cells (MCF-10A) were cultured in DMEM/F12 (Gibco, United States) supplemented with 5% horse serum, 10 μg/ml insulin, 20 ng/ml EGF (epidermal growth factor), 100 ng/ml cholera toxin and 0.5 μg/ml hydrocortisone. All cells were cultured in a humidified atmosphere of 5% CO_2_ and 95% air at 37°C. The cells were purchased from The Type Culture Collection of Chinese Academy of Sciencess, Shanghai, China. Cells from passages 3–5 were used for all experiments.

### Recombinant Protein Expression and Purification

The full-length wild-type p53 and the full-length p53 mutant (p53^R280K^) were expressed in *Escherichia coli* Transetta (DE3) (Transgene BioTech, China). The wild-type p53 gene was cloned into the pGEX-6p-2 vector (GE Life Sciences, United States) with sequence-specific primers (5′-ATT*GGATCC*ATGGAGGAGCCGCAG TCA-3′ and 5′-TTA*CTCGAG*TCAGTCTGAGTCAGGCCCT-3′). The mutant p53 (p53^R273H^ and p53^R280K^) was generated using a Fast Mutagenesis System Kit (Transgene BioTech, China) according to the manufacture’s instructions. Briefly, two truncated p53^R273H^ or p53^R280K^ sequences were amplified with sequence-specific primers (p53^R273H^, 5′-GTCCGTGTTTGTACGTGGAGTTTCGACAAG-3′ and 5′-GAGGTG CATGTTTGTGCCTGGACCGGCGCA-3′; p53^R280K^, 5′-T TTCCCAGGACAGGCA CAAACACGCACCTC-3′ and 5′-TTTGTGCCTGTCCTG GGAAAGACCGGCG CA-3′). Then the two truncated sequences were used a templates for PCR using p53-specific primers. The mutant p53^R273H^ or p53^R280K^ was cloned into the pGEX-6p-2 vector. The clones were confirmed by sequencing.

To explore the interaction between p53 protein domains and a compound, the wild-type p53 DNA binding domain (DBD) and DBD of mutant p53 (p53^R273H^ and p53^R280K^) were expressed in *Escherichia coli* Transetta (DE3). The genes were cloned into pET-Sumo plasmid that has a 10 KD Sumo tag (GE Life Sciences, United States) with sequence-specific primers (p53-DBD, 5′-AACGGATCCCAGAAAACCTACCAG GGCAGC-3′ and TAGCTCGAGCCCTTTCTTGCGGAGATTCTC-3′). The wild-type p53 N-terminal domain (NTD) and C-terminal domain (CTD) were expressed in *Escherichia coli* Transetta (DE3). The genes were cloned into the pET-28a plasmid that has a His tag and a T7 tag (about 3KD) (GE Life Sciences, United States) with sequence-specific primers (p53-NTD, 5′-ATCGGATCCATGGAGGAGCCG CAGTCAGATC-3′ and 5′-CGTCTCGAGGGAAGGGACAGAAGATGACAG; p53-CTD, 5′-AATGGATCCGAGCCTCACCACGAGCTGCCC-3′ and 5′-TCACTC GAGTCAGTCTGAGTCAGGCCCTTC-3′). The clones were confirmed by sequencing.

The recombinant bacteria were induced with 0.5 mM IPTG (isopropy-β-D-thiogalactoside) when OD_600_ (optical density at 600 nm) reached 0.5–0.6, followed by culture at 16°C for 20 h. The harvested bacteria were resuspended in binding buffer (50 mM Tris-Cl pH8.0, 300 mM NaCl, 0.1 mM PMSF) and sonicated for 30 min. Subsequently the supernatant was incubated with glutathione sepharose resin (GE Life Sciences, United States) at 4°C for 3 h. The recombinant protein was eluted by elution buffer (10 mM reduced glutathione, 20 mM Tris-HCl, pH8.0). The purity of the protein was resolved by SDS-PAGE (polyacrylamide gel electrophoresis).

### Extraction of Metabolites From GVE2-Infected *Geobacillus* sp. E263

The deep-sea thermophile *Geobacillus* sp. E263 (China General Microbiological Culture Collection Center accession no. CGMCC1.7046) was cultured at 60°C in TTM medium (0.2% NaCl, 0.4% yeast extract, 0.8% tryptone; pH 7.0) supplemented with 25 mM MgSO_4_. Then the E263 strain was infected with its bacteriophage GVE2 at log phage (*OD*_600_ = 0.4) and further cultured for 24 h. The crude metabolites were extracted from GVE2-chellenged *Geobacillus* sp. E263 with methanol at 4°C for three times. The supernatant was obtained by filtration and evaporated to dryness on a rotary evaporator under reduced pressure. The evaporated metabolites were dissolved in DMSO (dimethyl sulphoxide) at 5% final concentration and stored at −80°C.

### Screening of Metabolites Bound to p53 Protein From GVE2-Infected *Geobacillus* sp. E263

The recombinant wild-type p53 and p53 mutant protein were incubated with the crude metabolites of GVE2-chellenged *Geobacillus* sp. E263 at 4°C for 3 h. GST was used as a control. Subsequently, the proteins were applied to a Sephadex G-50 size exclusion chromatography column (GE Life Sciences, United States) in a preparative HPLC (High Performance Liquid Chromatography) system. The protein elution peak was detected by absorbance at 280 nm wavelength, collected and subjected to the extraction of the metabolite bound to the proteins with methanol.

### Identification of the Compound Bound to the Mutant p53 Protein

The compound bound to the mutant p53 protein or the standard compound synthesized by Yuhao Chemical Company (Hangzhou, China) was subjected to silylation derivatizaion prior to GC-MS (gas chromatography mass spectrum) analysis. GC-MS analysis was performed on a DSQ II Quadruppoles mass spectrometry (Thermo Electron Corporation, United States). Briefly, the compound was treated with 20 μL of methoxyamine hydrochloride solution at 80.8 mM in pyridine and then reacted for 125 min at 35°C. The sample was silylated for 125 min at 35°C upon the addition of 32 μL methyl-trimethylsilyl-trifluoroacetamide (MSTFA) (Sigma, United States). The dicyclohexylphthalate was used as the derivatization standard. For each run, 2 μL of derivatized sample was injected at 60°C. During the course of the run, the temperature of the column was held at 60°C for 4 min, then ramped to 200°C at a rate of 5°C/min and held at 200°C for 1 min. Subsequently the temperature was increased to 280°C at a rate of 18°C/min, and held for another 8 min. The mass spectrometry was operated at electron impact mode at 70 eV. The source temperature was set at 200°C. Full-scan mass spectra were acquired from 40 to 600 m/z with a scan rate of five times per second using Xcalibur version 2.0.7. The compound was identified by searching the NIST (National Institute of Standards and Technology, United States) mass spectral libraries by NIST MS search software (version 2.0) (Agilent Technologies, United States). After its identification, the compound and its derivatives used in the following experiments were synthesized by Yuhao Chemical Company (Hangzhou, China).

### Screening of Proteins Bound to the Identified Compound

The identified compound was labeled with biotin using EZ-Link^TM^ Sulfo-NHS-LC- Biotinylation Kit (Thermo Fisher Scientific, United States) according to the manufacutre’s protocol. MDA-MB-231 cells (10^7^) were lysed in lysis buffer (50 mM Tris-HCl, 137 mM NaCl, 1 mM EDTA, 1% Triton X-100, 1 mM phenylmethanesulfonyl fluoride, pH8.0) at 4°C for 2 h., followed by incubation with biotin or biotin-labeled compound at 4°C for 4 h. Subsequently, the mixture was incubated with streptavidin agarose at 4°C for 4 h. The proteins bound to the compound were analyzed by SDS-PAGE (Sodium dodecyl sulfate polyacrylamide gel electrophoresis) and stained with Coomassie brilliant blue.

### Cell Viability Assay

Cell viability assay was conducted using a CellTiter 96^®^ AQueous One Solution Cell Proliferation Assay Kit (Promega, United States). Briefly, 5,000 cells were seeded onto a 96-well plate and incubated at 37°C overnight. The cells at 60% confluence were incubated a compound at different concentrations or DMSO for 48 h. Subsequently 20 μl of MTS [3-(4, 5-dimethylthiazol-2-yl)-5-(3-carboxymethoxyphenyl)-2-(4-sulfophenyl)-2H-tetrazolium, inner salt] reagent was added into the cells. Three hours later, the absorbance at 450 nm of samples was recorded using a GloMax 96 microplate reader (Promega, United States).

### Detection of Apoptosis by Measuring Caspase3/7 Activity

Cells were plated into a 96-well plate at 5,000 cells/well. After incubation overnight at 37°C, the identified compound was added into the cells. The cells were incubated for 24 h and then 100 μl Caspase-Glo reagent (Promega, United States) was added, followed by incubation for 1 h at room temperature. The fluorescence of cells was determined using a GloMax 96 microplate reader (Promega, United States).

### Apoptosis Detection by Flow Cytometry

Cells were incubated treated with the identified compound for 5 h. Then the cells were harvested and washed with cold PBS. The cells were resuspended in 1 × binding buffer (BD Pharmingen, United States) at a concentration of 1 × 10^6^ cells/ml. Subsequently Annexin V and PI (propidium iodide) (BD Pharmingen, United States) were added into the cells. After incubation for 15 min at 25°C in the dark, the cells were examined by flow cytometry.

### Detection of p53 Protein Status by Immunofluorescent Staining

The cells were seeded on a coverslip (Corning Life Sciences, United States) and then incubated at 37°C overnight. Subsequently the cells were fixed with 4% paraformaldehyde for 10 min at room temperature. The cell membrane was permealized with 0.5% Triton X-100 for 5 min to allow the entry of antibody into cells. Then the permealized cells were blocked with 1% BSA (bovine serum albumin) for 1 h at room temperature, followed by incubation with the primary antibody PAB1620 or PAB240 (Abcam, United States) at 4°C overnight. These antibodies can recognize the p53 protein status. PAB1620 and PAB240 are able to specifically recognize wild-type and mutated p53, respectively (Yu et al., 2012). Thereafter, the cells were incubated with the goat anti-mouse IgG Alexa Fluor 488 secondary antibody (Invitrogen, United States) for 1 h at room temperature. The nuclei were stained with DAPI (4′, 6-diamidino-2- phenylindole) for 10 min at room temperature. After washes with PBS, the immunofluorescence of cells was detected by laser scanning confocal microscope LSM700 (Zeiss, Germany).

### Transcriptional Activity Assay of p53

Breast cancer cells (MDA-MB-231, MDA-MB-468, and MCF-7) and non-cancer cells (MCF-10A) were seeded into a 6-well plate and then transfected with pp53-TA-luc plasmid (Beyotime Biotechnology, China) that contained a firefly luciferase controlled by a 20-bp p53 response element and an internal control pRL-TK plasmid (Promega, United States) which contained a renilla luciferase constitutively controlled by HSV-TK (herpes simplex virus- thymidine kinase) promoter using Lipofectamine 2000 (Life technologies, United States). At 6 h after transfection, the medium was replaced with fresh medium containing 0.1% DMSO (control) or 50 μM HBAP. Twenty-four hours later, the firefly luciferase and renilla luciferase activities of cells were determined by a dual luciferase reporter assay system kit (Promega, United States) according the manufactures’ instructions. The ratio of firefly luciferase activity to renilla luciferase activity represented the relative transcriptional activity of p53.

### Immunoblotting Analysis

Proteins were separated by SDS-PAGE and then transferred onto a nitrocellulose membrane (Milipore, United States). The membrane was incubated with blocking buffer [3% skim milk in TBST (Tris-buffered saline, 0.05% tween-20)] for 2 h at room temperature. Subsequently the membrane was incubated with a primary antibody overnight at 4°C, followed by incubation with HRP (horseradish peroxidase)-conjugated secondary antibody (Bio-Rad, United States) for 2 h at room temperature. After washes with TBST for three times, the membrane was incubated with ECL (enhanced chemiluminescence substrate) (PerkinElmer, United States) for 5 s. The proteins were detected by ChampChemi 610 (Sage Creation science, Beijing, China).

### Expression of p53 in H1299 Cells

H1299 cells were transfected with pcDNA3.1 plasmid expressing wild-type p53 or mutant p53 (p53^R280K^ and p53^R273H^) using lipofectamine 2000 (Invitrogen, United States). At 48 h after transfection, the cells were collected. Then total RNAs were extracted using total RNA rapid extraction kit (Generay Biotechnology, China). The cDNA was reversely transcribed with ReverTra Ace qPCR RT kit (Toyobo, Japan). To examine the expression level of p53, quantitative real-time PCR was conducted at 95°C for 5 min, followed by 40 cycles at 95°C for 15 s and 60°C for 30 s. Quantitative real-time PCR reaction mixture (10 μl) contained 5 μl 2 × SYBR Premix Ex Taq (Takara, Japan), 0.4 μl p53-specific primers (5′-ATCTACTGGGACGGAACAGC-3′ and 5′-GCGGA GATTCTCTTCCTCTG-3′) and 1 μl cDNA template. The *p53* mRNA level was normalized to the *GAPDH* (glyceraldehyde-3-phosphate dehydrogenase) gene expression level.

### ITC Assay

ITC (isothermal titration calorimetry) assay with recombinant expressed proteins and the identified compound was performed in a VP-ITC system (GE Healthcare, United States). Proteins were dissolved in buffer A (50 mM HEPS-HCl, 300 mM NaCl, 5% DMSO, pH 8.0) at a final concentration of 100 μM. Then, 1.42 mL of the protein solution was loaded into the VP-ITC cell. The identified compound (1 mM) dissolved in buffer A and was added to the ITC syringe. Titration was conducted at 30°C with the reference power set to 10. Thirty successive injections of 10 μl compound were carried out into the cell, in a 120 s interval for each titration and 130 rpm stirring speed. ITC data were collected using the Microcal Origin and analyzed after subtracting a reference data. The injection peaks were integrated using a single-site binding model employed by the Origin software (GE Healthcare, United States).

### Tumorigenicity in Nude Mice

Breast cancer cells (MDA-MB-231) were cultured in Leibovitz’s L15 medium (Gibco, United States) and collected after reaching 80% confluence. Then, the cells (5 × 10^6^ cells/ml) were subcutaneously injected into nude non-obese female mice that had immunodeficiency combined with severe diabetes to induce tumor growth. Two weeks later, the mice were intraperitoneal injected with 50 μl of the identified compound dissolved in 5% DMSO at a concentration of 10 mg/Kg or 5% DMSO in corn oil as a control. The identified compound and DMSO were injected into mice every 2 days. Control and drug-treated groups contained four animals. Tumor volumes were measured every 2 days. Eight weeks later, the mice were euthanized and their tumors were evaluated. Animal experiments were approved by the Ethics Commitee of the Animal Experiment Centre at Zhejiang University, China.

### Immunohistochemistry

The solid tumors were fixed using 4% paraformaldehyde at 4°C overnight and paraffin-embedded. Then they were sectioned at 4-μm thickness. After H & E (hematoxylin-eosi) staining, the sections were incubated with the antibody against cleaved caspase 3 (Cell Signaling Technology, United States) at 4°C overnight. The sections were rinsed with PBST (PBS+0.5% Tween-20) and then incubated with HRP (horseradish peroxidase)-coupled secondary antibody (Bio-Rad, United States) at room temperature for 2 h and, followed by fluorescence detection after DAB (Diaminobenzidine) (Agilent Technology, United States) addition. The sections were observed under a DS-U3 microscopy (Nikon, Japan).

### Toxicological and Pharmacologic Assays of HBAP in Mice

For pharmacokinetic evaluation, mice were injected intraperitoneally with 500 nM HBAP diluted in corn oil (Aladdin, Shanghai, China) per mouse. Corn oil was injected alone as a control. After 0.5, 3, and 5 h of the injection, the HBAP content in mouse blood was measured by liquid chromatography high-resolution mass spectrometry (LC-MS). For toxicologial assessment, the mice were injected with 500 nM HBAP daily over a 2 weeks period. On the 14th day, the mice blood of was collected in a EDTA (ethylenediaminetetraacetic acid)-containing tube. After centrifugation at 500 × g (4°C) for 2 min, cells were collected and subjected to white and red blood cell count using COBASC 311 (Roche Diagnostics, Switzerland). Mice were maintained at room temperature for both evaluations.

### Statistical Analysis

All experiments were biologically repeated for three times and the data were analyzed using a one-way analysis of variation. The statistical significance between treatments was determined with Student’s *t-*test.

## Results

### A Deep-Sea Thermopile-Derived Compound Can Specifically Bind to the Mutated p53 Protein

**FIGURE 1:**
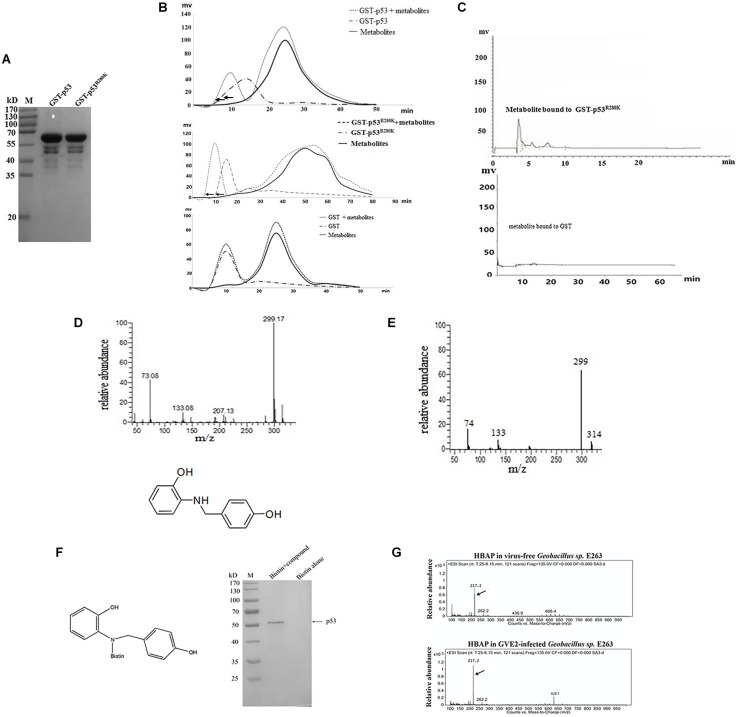
A deep-sea thermopile-derived compound can specifically bind to the mutated p53 protein. **(A)** Recombinant expression of full-length wild-type p53 and mutant p53^R280K^ as GST-fusion proteins in *E. coli*. Purified proteins were analyzed using SDS-PAGE with Coomassie blue staining. M, protein marker. **(B)** Identification of metabolites bound to the wild-type p53, mutant p53 protein or GST protein. Purified recombinant wild-type p53 and mutant p53 proteins were incubated with the metabolites extracted from the GVE2-challenged *Geobacillus* sp. E263 and applied to a Sephadex G-50 size exclusion chromatography column in a preparative HPLC system. The protein elution was monitored by absorbance at 280 nm. GST was used as a control. **(C)** Analytical HPLC analysis of the metabolite bound to the mutant p53 protein (p53^R280K^). **(D)** GC-MS analysis and identification of the metabolite bound to the p53^R280K^ protein. After searching the NIST (National Institute of Standards and Technology, United States) mass spectral libraries using the obtained GC-MS spectra, the compound was identified as the 2-[(4-hydroxybenzyl) amino] phenol (HBAP). **(E)** GC-MS spectra of the standard HBAP. **(F)** The binding specificity of HBAP to the mutant p53 protein. The HBAP compound was coupled with biotin and the MDA-MB-231 breast cancer cell lysate was incubated with biotin or biotin-labeled compound. The proteins bound to the HBAP were analyzed using SDS-PAGE with Coomassie blue staining. The bound protein is indicated by the arrow. **(G)** Virus infection influence on the HBAP production. *Geobacillus* sp. E263 was challenged by GVE2 bacteriophage. Twenty-four hours later, the metabolites extracted from GVE2-infected E263 were analyzed by GC-MS. Arrows indicated the HBAP compound.

To identify metabolites from deep-sea thermophile organisms capable of binding to mutant p53 protein, the wild-type p53 and the mutant p53^R280K^ were expressed as GST-fusion proteins in *E. coli* and then purified (Figure 1A). After purification, recombinant GST-p53 and GST-p53^R280K^ fusion proteins were incubated with the metabolites extracted from GVE2-challenged *Geobacillus* sp. E263 and applied to a Sephadex G-50 size exclusion chromatography column in a preparative HPLC system. The absorbance GST- p53^R280K^ peak at 280 nm in the presence of metabolites was found significantly shifted compared to that of the GST- p53^R280K^ alone (Figure 1B). These data indicate that some metabolites bound to the p53^R280K^ since the displacement in the 280 nm absorbance peak may be the result of conformational changes in the p53^R280K^ structure after metabolites binding. In addition, the absorbance peak at 280 nm of the GST-p53 and GST control did not change in the metabolites presence (Figure 1B), showing that no metabolite bound to these proteins. These data indicated that some metabolites specifically bound to the mutant p53 protein (p53^R280K^). Then, the metabolites were collected bound to the protein at the 280 nm absorbance peak, separated from it and subjected to separation by analytic HPLC for their identification. Analytic HPLC results showed that there was a unique metabolite bound to the GST-p53^R280K^ fusion protein, while there was no metabolite bound to GST-p53 or GST control (Figure 1C). These data revealed that a specific metabolite can bind to the p53^R280K^ protein. GC-MS analysis identified that the metabolite bound to GST-p53^R280K^ fusion was 2-[(4-hydroxybenzyl) amino] phenol (HBAP) (Figure 1D). The GC-MS spectra of the standard HBAP were consistent with those obtained from the separated metabolite bound to the GST-p53^R280K^ fusion protein (Figure 1E), reiterating the identified interaction of HBAP with the p53^R280K^ protein.

To identify the specificity of HBAP binding to the mutant p53 protein, this compound was coupled with biotin and then an affinity assay was performed using biotin labeling. SDS-PAGE results indicated that the HBAP can bind to the GST-p53^R280K^ fusion protein but not to the control (biotin alone) (Figure 1F), showing the specificity of HBAP binding to the mutant p53 protein. To explore the influence of virus infection on HBAP production, *Geobacillus* sp. E263 was challenged with GVE2. GC-MS analysis showed that the HBAP abundance was increased by GVE2 infection compared to the control (Figure 1G). These results indicated that virus challenge can induce the production of bacterial metabolites associated with apoptosis. The above findings revealed that the HBAP compound, derived from deep-sea virus-challenged thermophile *Geobacillus* sp. E263, interacted specifically with the mutant p53 protein (p53^R280K^).

### The HBAP Compound Induces p53-Dependent Apoptosis of p53-Mutated Breast Cancer Cells

**FIGURE 2:**
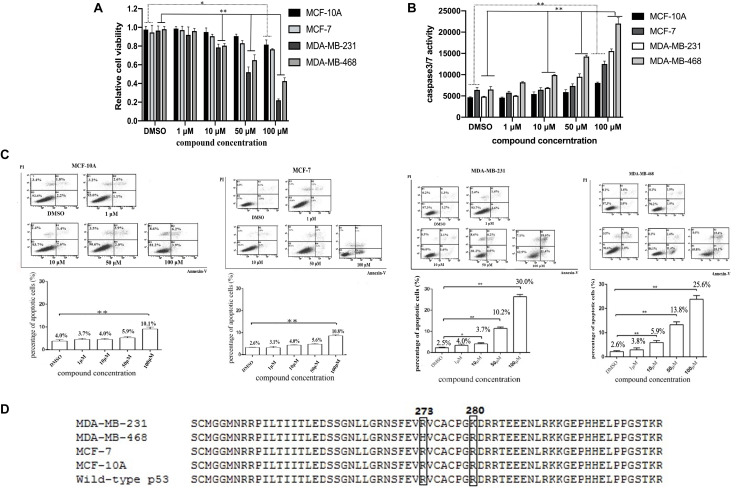
The HBAP compound induces p53-dependent apoptosis of p53-mutated breast cancer cells. **(A)** HBAP compound influence on the proliferation of breast cancer cell lines (MCF-7, MDA-MB-231 and MDA-MB-468) and normal breast cells (MCF-10A). Forty-eight hours after treatment, the cell proliferation was examined using a CellTiter 96^®^ AQueous One Solution Cell Proliferation Assay Kit. **(B)** HBAP effects on the caspase 3/7 activity of breast cancer cell lines and normal breast cells. Twenty-four hours after treatment, the caspase3/7 activity of the cells was evaluated. **(C)** Early apoptosis detection in breast cancer cell lines and normal breast cells. Five hours after treatment, the cells were analyzed by flow cytometry using Annexin V/PI staining. **(D)** Sequencing of the *p53* sequence in breast cancer cell lines and normal breast cells. The *p53* mutations are highlighted within the boxes. For the experiments on **(A–C)**, all cell lines were treated with different concentrations of HBAP. For the experiments on **(B,C)**, DMSO as used as a control. In all panels, data were shown as mean ± SD of three biological repeats (^∗^*p* < 0.05; ^∗∗^*p* < 0.01).

To assess the HBAP effects on cancer cell apoptosis, the proliferation of normal breast cells (MCF-10A) and three breast cancer cell lines (MDA-MB-231, MDA-MB-468, and MCF-7) treated with different concentrations of this compound was investigated. The results showed that the proliferations of cancer cell lines MDA-MB-231 and MDA-MB-468 was significantly supressed by HBAP at lower concentrations, while the proliferation of MCF-7 and MCF-10A cells was slightly inhibited at 100 μM HBAP (Figure 2A). The caspase 3/7 activity of the compound-treated cancer cells was examined. The results indicated that the caspase 3/7 activity of the MDA-MB-231 and MDA-MB-468 cancer cell lines treated with low HBAP concentrations was significantly enhanced compared to the control (Figure 2B). However, the caspase 3/7 activity of MCF-7 and MCF-10A cells was increased only in treatments with a HBAP high concentration (Figure 2B). These data are consistent with those of cell proliferation analysis. Flow cytometry analysis provided similar results (Figure 2C). These data revealed that HBAP can promote apoptosis of two breast cancer cell lines (MDA-MB-231 and MDA-MB-468) in a p53-dependent manner.

To determine why the HBAP compound can induce apoptosis of MDA-MB-231 and MDA-MB-468 cells, but not of MCF-7 and MCF-10A, the p53 gene of these cell lines was sequenced. Sequencing data showed that the p53 gene is mutated in the MDA-MB-231 (p53^R280K^) and MDA-MB-468 (p53^R273H^) cell lines, but not in the MCF-10A and MCF-7 cells (Figure 2D). These results demonstrate that the HBAP can only induce apoptosis of p53-mutated breast cancer cells.

### HBAP Activates the Transcriptional Activity of the Mutant p53 by Restoring Its Function to That of Wild-Type p53

**FIGURE 3:**
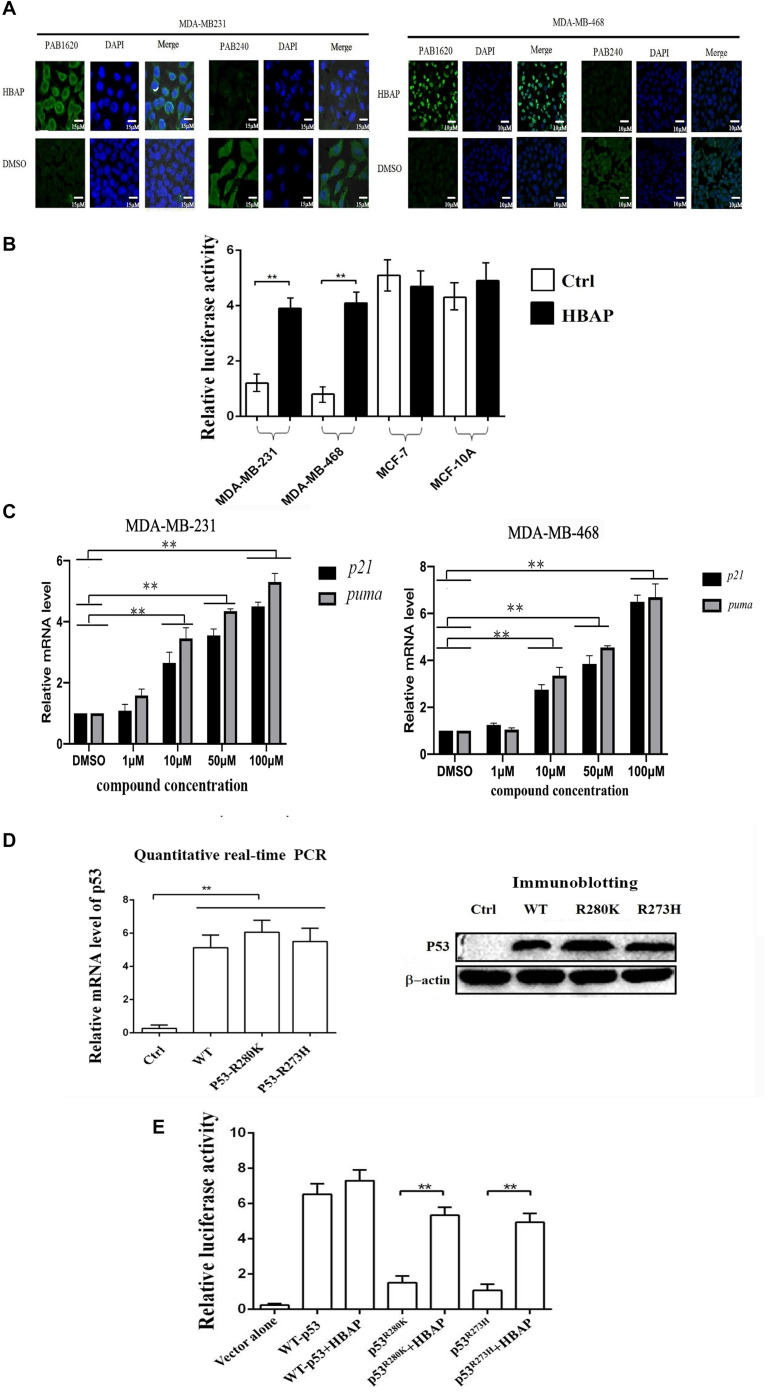
HBAP activates the transcriptional activity of the mutant p53 by restoring its function to that of wild-type p53. **(A)** Restoration of the mutant p53 function to that of wild-type p53 induced by HBAP. MDA-MB-231 (p53^R280K^) and MDA-MB-468 (p53^R273H^) cells were treated with HBAP or DMSO (control). Then, the cells were incubated with PAB1620 (recognizing wild-type p53 conformation) and PAB240 (recognizing mutant p53 conformation) antibodies, respectively. The nuclei were stained with DAPI. **(B)** The influence of HBAP on the transcriptional activity of p53 in cells. The p53-mutated breast cancer cells (MDA-MB-231 and MDA-MB-468) and the cells containing wild-type p53 (MCF-7 and MCF-10A) were treated with HBAP and subjected to p53 transcriptional activity assays. DMSO-treated cells were used as controls. **(C)** The impact of HBAP on the expressions of p21 and PUMA (p53 upregulated modulator of apoptosis) in MDA-MB-231 and MDA-MB-468 cells. The cells were treated with HBAP at various concentration or DMSO as a control. The expression levels of *p21* and *puma* were examined by quantitative real-time PCR (^∗∗^*p* < 0.01). **(D)** Expression of wild-type p53 or mutant p53 in p53-null lung cancer cells (H1299). The plasmid expressing wild-type p53 or mutant p53 (p53^R280K^ or p53^R273H^) was transfected into H1299 cells. The expression level of wild-type or mutant p53 was examined by quantitative real-time PCR or immunoblotting analysis. The cells transfected with vector only were used as controls. **(E)** The effects of HBAP on the transcriptional activity of mutant p53. HBAP (50 μM) was incubated with H1299 cells expressing wild-type or mutant p53. At 24 h after incubation, the cells were subjected to transcriptional activity assay of p53. In all panels, the statistical significances between treatments were indicated with asterisks (^∗∗^*p* < 0.01).

To reveal the mechanism of HBAP inducing apoptosis of p53-mutated breast cancer cells, the influence of HBAP on the conformation of mutant p53 was characterized in MDA-MB-231 and MDA-MB-468 cells, in which p53 was mutated (R273H and R280K). The results of immunoflourescence staining indicated that HBAP-treated MDA-MB-231 and MDA-MB-468 cells were labeled by PAB1620, an antibody specifically recognizing the conformation of wild-type p53 protein, but not by PAB240, an antibody specifically recognizing the conformation of mutated p53 protein (Figure 3A). In contrast, the DMSO-treated cells were stained by PAB240, but not by PAB1620 (Figure 3A). These data revealed that HBAP could restore mutant p53 to the level similar to wild-type p53 protein in p53-mutated breast cancer cells.

To evaluate the HBAP influence on the p53 transcriptional activity, the p53-mutated cell lines (MDA-MB-231 and MDA-MB-468) and the wild-type p53-containing cells (MCF-7 and MCF-10A) were transfected with pp53-TA-luc plasmid and the internal control pRL-TK plasmid, followed by treatment with HBAP or DMSO. The results indicated that HBAP significantly increased the luciferase activity of p53-mutated cells compared to the control (Figure 3B). However, HBAP had no influence on the transcriptional activity of wild-type p53 (Figure 3B). At the same time, HBAP significantly increased the expression levels of the target genes [p21 and PUMA (p53 upregulated modulator of apoptosis)] of p53 in p53-mutated breast cancer cells (MDA-MB-231 and MDA-MB-468) (Figure 3C). These findings indicated that HBAP could activate the transcriptional activity of p53 by binding to the target DNAs in p53-mutated cancer cells.

To validate whether HBAP is capable of activating the transcriptional activity of mutated p53 protein, the plasmid expressing wild-type or mutant p53 was transfected into p53-null lung cancer cells (H1299). Quantitative real-time PCR and immunoblotting analyses showed that the wild-type p53 and mutant p53 (p53^R280K^ and p53^R273H^) were expressed in p53-null cancer cells (Figure 3D). Transcriptional activity assays of p53 indicated that HBAP significantly increased the transcriptional activity of mutant p53, but not of wild-type p53 (Figure 3E).

Taken together, these results revealed that the HBAP compound can activate the transcriptional activity of mutant p53 by restoring its function as that of wild-type p53.

### The DNA-Binding Domain of the Mutant p53 Protein Interacts With the Hydroxyl Groups of the HBAP Compound

**FIGURE 4:**
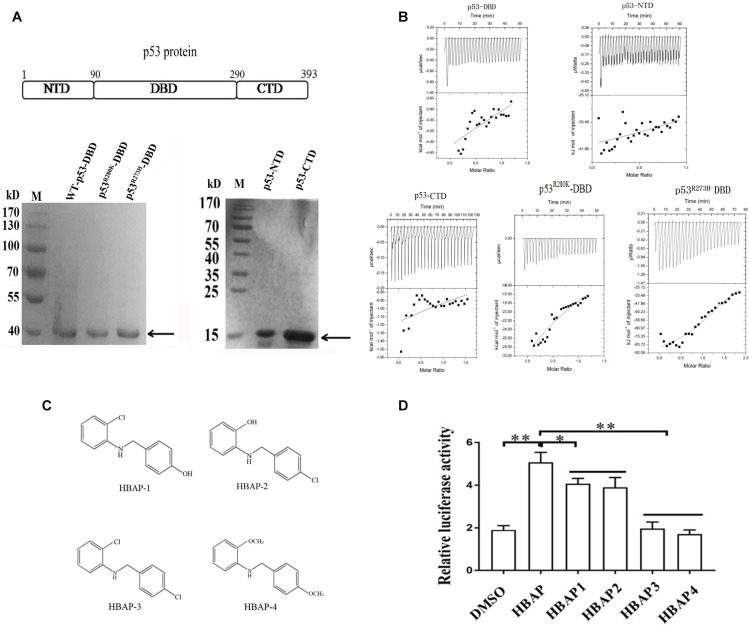
The DNA-binding domain of the mutant p53 protein interacts with the hydroxyl groups of the HBAP compound. **(A)** Recombinant expression as GST-fusion proteins in *E. coli* and purification of NTD, DBD and CTD domains of wild-type p53 protein and DBD domains of mutant p53 (p53^R280K^ and p53^R273H^). Target proteins were indicated by the arrows. **(B)** Isothermal calorimetric data for HBAP interactions with NTD, DBD and CTD domains of wild-type p53 and DBD domains of mutant p53 (p53^R280K^ and p53^R273H^). **(C)** Structures of HBAP derivatives. **(D)** Effects of HBAP and its derivatives on the p53 mutant transcriptional activity in breast cancer cells (MDA-MB-468). The breast cancer cells were transfected with both pp53-TA-luc plasmid and pRL-TK plasmid. Twelve hours after transfection, HBAP or its derivatives were to the cells. After 24 h, the relative luciferase activity was measured (^∗^*p* < 0.01; ^∗∗^*p* < 0.01).

The three domains of the wild-type p53 (NTD, DBD and CTD) and the DBD domains of mutant p53 (p53^R280K^ and p53^R273H^) were expressed as GST-fusion proteins in *E. coli*, purified (Figure 4A) and subjected to ITC assays with HBAP to determine which of these domains is able to bound this compound. ITC results revealed that HBAP bound only to GST-DBD of the mutant p53 (p53^R280K^ and p53^R273H^), showing no affinity for NTD, CTD and DBD domains of wild-type p53 (Figure 4B). These data indicated that the HBAP interacted specifically with the DNA binding domain of the mutated p53 protein.

To characterize the HBAP functional groups that interacted with DBD of the mutant p53, four HBAP derivatives were synthesized (Figure 4C) and added to breast cancer cells (MDA-MB-468) that contained a mutant p53 (p53^R273H^). Dual luciferase assays results showed that the p53 transcriptional activity decreased significantly when one of the hydroxyl functional groups was replaced by chloride (Figure 4D). In the absence of hydroxyl functional groups, the p53 transcriptional activity was the same as that of the control (DMSO) (Figure 4D). These data indicate that the HBAP hydroxyl groups of are necessary to HBAP interaction with DBD domain of mutant p53 and to restore its function as that of wild-type p53.

### HBAP Suppresses Tumor Proliferation in Mice *in vivo*

**FIGURE 5:**
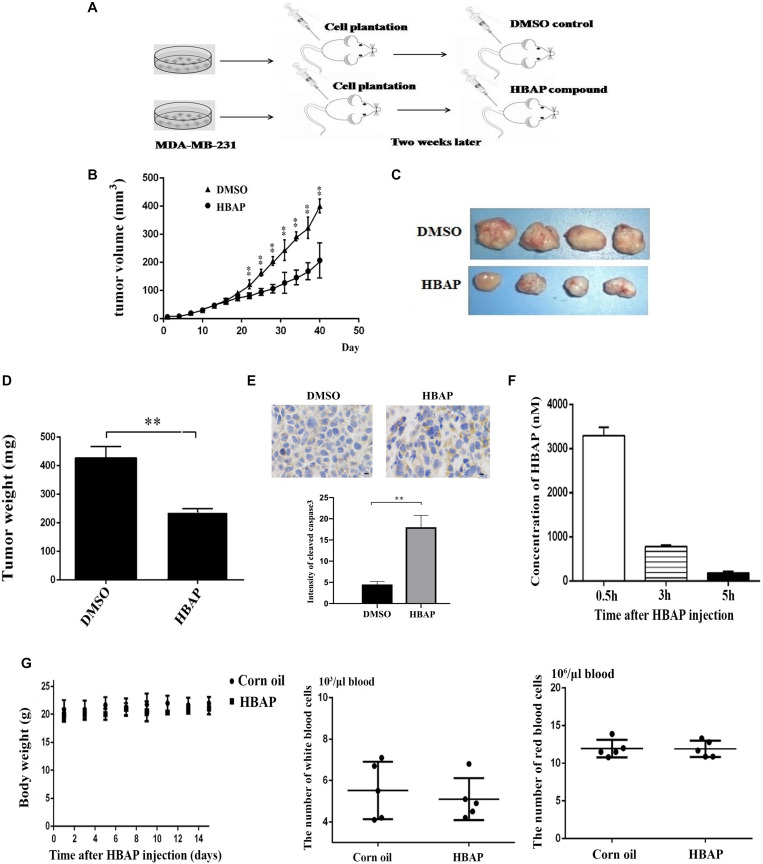
HBAP suppresses tumor proliferation in mice *in vivo*. **(A)** The flow chart of xenograft mouse tumor experiment. Breast cancer cells (MDA-MB-231) were subcutaneously injected into female non-obese diabetic severe combined immune-deficient mice to induce tumor growth. Two weeks later, the mice were subcutaneously injected with HBAP (10 mg/Kg pre mouse) or 5% DMSO (in corn oil). The compound or DMSO was injected into mice every 2 days. For each treatment, four mice were used. Eight weeks later, the nude mice were euthanized and their tumors were evaluated. **(B)** The effects of HBAP on tumor growth in nude mice. Breast cancer cells were injected into nude mice. Two weeks later, HBAP or DMSO (a control) was injected into mice. The tumor volumes were examined every 3 days. **(C)** Solid tumors from xenograft mice treated with HBAP or DMSO. **(D)** Solid tumor weight of mice treated with DMSO or HBAP. **(E)** Immunohistochemical analysis of solid tumors. The solid tumors from xenograft mice treated with HBAP or DMSO were stained with cleaved-caspase 3 antibody. Yellow represented the cleaved-caspase 3 and blue indicated nuclei. Four solid tumors were used to stain cleaved caspase-3. Three fields were analyzed for each tumor. The quantitative data were also presented (^∗∗^*p* < 0.01). Scale bar, 10 μm. **(F)** The content of HBAP in the blood of HBAP-treated mice (*n* = 3). HBAP was quantified with liquid chromatography high-resolution mass spectrometry. **(G)** The influence of HBAP on mouse body weight, and the numbers of white blood cells and red blood cells. Corn oil was included in the assays as a control. In all panels, the statistical significance between treatments was indicated with asterisks (^∗∗^*p* < 0.01).

Xenograft mouse tumor experiments were conducted using human breast cancer cells MDA-MB-231 to explore the HBAP effects on tumorigenesis *in vivo* (Figure 5A). Breast cancer cells were transplanted into nude mice and 2 weeks after the xenograft, the mice were treated with HBAP. The results showed that the tumor growth was significantly decreased by HBAP compared to the control (DMSO) (Figure 5B). The solid tumor size and weight of the HBAP-treated mice were significantly smaller than those of the control (Figures 5C,D). These findings indicate that HBAP can inhibit tumor growth *in vivo*. The apoptotic activity of solid tumors from the HBAP-treated xenograft mice was also examined. Immunohistochemical data revealed that the solid tumors apoptotic activity from HBAP-treated mice was significantly increased compared to that of the control (Figure 5E). These data show that HBAP can inhibit tumor growth *in vivo* by inducing tumor cell apoptosis.

The toxicological and pharmacological effects of HBAP were examined after this compound was injected into mice by assessing the HBAP content in blood, body weight and number of red and white blood cells. Pharmacological assays results indicated that the HBPA concentration in mice blood reached its maximum 0.5 h after the HBAP injection and its minimum after 5 h (Figure 5F). Toxicological data showed that HBAP had no effect on the body weight and on the red and white blood cell number compared to the control (Figure 5G). These results indicate that HBAP is toxicity-free.

## Discussion

P53, a well-known tumor suppressor, acts as a transcriptional factor by binding to specific DNA sequences and transactivating the expressions of various target genes involved in apoptosis and cell-cycle arrest (Joerger and Fersht, 2016). The *p53* gene is frequently mutated in many human cancers, such as breast cancer, lung cancer and colon cancer (Desantis et al., 2015). Human cancer patients (*N* = 19,262) from the International Agency for Research on Cancer (IARC) TP53 database indicate that most mutations of p53 are located in the DBD domain (Amelio et al., 2016). These mutations not only abrogate its tumor suppressive activity but also lead to a gain in function by promoting tumor cell proliferation (Freed-Pastor and Prives, 2012). As mutations in p53 occur in human cancers and are critical to tumorigenesis, the restoration of mutant p53 to the wild-type has been explored by several studies in recent years. Restoring endogenous p53 expression in mice by temporally regulating p53 expression using Cre-*lox*P-based strategy has been reported to lead to the regression of autochthonous lymphomas and sarcomas (Ventura et al., 2007). Some compounds have been found capable of restoring the mutant p53 to exert its wild-type function and lead to cancer progression supression. MIRA-1 is a small molecular compound screened from the National Cancer Institute (NCI) compound libraries that can reactivate the mutant p53 DNA binding activity and induce tumor cells to apoptosis by restoring the mutant p53 transcriptional activity (Bykov et al., 2005). The WR1065 compound is capable of changing the conformation of the temperature-sensitive mutant p53 to that of the wild-type p53 at 37°C and thus stimulating the mutant p53 DNA binding activity to increase the expression of p53 downstream genes (North et al., 2002). Currently, the compounds that can restore the mutant p53 function and conformation to those of wild-type p53 are obtained from artificial synthetic compound libraries. To date, no compound with these effects has been obtained from natural products.

In this study, we show that the HBAP compound obtained from virus-challenged *Geobacillus* sp. E263, a deep-sea hydrothermal vent thermophile organism, can restore mutant p53 function to that of wild-type p53, leading to apoptosis of breast cancer cells. Remarkably, HBAP had no effect on apoptosis of non-cancerous breast cells. In fact, HBAP were patented many years ago (Renth et al., 1979; Walensky, 2012), which could modulate cell death, cell division and cell differentiation. However, the underlying mechanism of HBAP regulating cell death has not been explored. Breast cancer has been the leading cause of cancer death in woman worldwide and in which mutated p53 is associated with a poor prognosis (Desantis et al., 2015). Mutations in amino acid residues 200–300 of p53 protein, a region located within the DBD domain, occur with great frequency in breast cancer (Grawenda et al., 2015). No compound capable of reactivating the function of mutant p53 for that of wild-type p53 in breast cancer has been identified. Our study demonstrated that HBAP recovered the normal transcriptional activity of the mutant p53 by binding to the DBD domain in breast cancer cells. Thus, HBAP may be a good candidate for breast cancer therapy with mutated p53. In addition, the metabolites from deep-sea virus-challenged thermophiles can be a promising source of anticancer drugs.

Deep-sea hydrothermal vents host organisms that do not derive their energy from the sun, but from bacterial oxidation of chemicals in the vent fluids, particularly hydrogen sulfide, making them special ecosystems very different from the terrestrial ones (Rogers et al., 2012). Chemolithoautotrophic microorganisms exploit chemical energy for their growth (He et al., 2017). These chemosynthetic thermophiles synthesize organic matter and provide primary nutrients for the entire ecosystem, thus serving as the basis of the food chain in deep-sea hydrothermal ecosystems (He et al., 2017). Vent ecosystems has been reported to be rich in bacteriophages (Wei and Zhang, 2009; He et al., 2017), which significantly alter the metabolism of bacteria by infecting them (Jin et al., 2015). Regarding metabolism disorder, there is a relationship between human tumorigenesis and viral infection in bacteria. Therefore, the bacteriophage-challenged thermophiles of deep-sea vents may produce special metabolites for screening of anti-tumor drugs due to the deep-sea vent ecosystems specificity. Our findings demonstrated that the metabolites of virus-infected microorganisms from deep-sea vents deserve to be further explored.

## Data Availability Statement

The original contributions presented in the study are included in the article/supplementary material, further inquiries can be directed to the corresponding author.

## Ethics Statement

The animal study was reviewed and approved by the Animal Experiment Centre of Zhejiang University, China.

## Author Contributions

XZ conceived, designed research, and revised the manuscript. CX and JZ collected the data, conducted the research, and wrote the initial manuscript. All authors read and approved the final manuscript.

## Conflict of Interest

The authors declare that the research was conducted in the absence of any commercial or financial relationships that could be construed as a potential conflict of interest.
